# Correlation between musculoskeletal mass and perfusion in patients with gastrointestinal malignancy: a preliminary study based on quantitative CT and CT perfusion

**DOI:** 10.1186/s12891-022-05288-8

**Published:** 2022-04-08

**Authors:** Rui Ji, Lin Zhang, Yongju Shen, Rui Tang, Yun Tu, Guangyu Tang, Jingqi Zhu

**Affiliations:** Department of Radiology, Shanghai Tenth People’s Hospital, Tongji University School of Medicine, 301 Middle Yanchang Road, Shanghai, 200072 China

**Keywords:** Bone mineral density, Muscular mass, Quantitative computer tomography, CT perfusion, Gastrointestinal malignancy

## Abstract

**Background:**

To investigate the correlation between musculoskeletal mass and perfusion using quantitative computer tomography (QCT) and CT perfusion (CTP) in patients with gastrointestinal malignancy.

**Methods:**

In this prospective study, 96 patients (mean age 66 years, range 25–90; 63.5% male) with gastrointestinal malignancy underwent QCT and CTP between May 2019 and February 2021. Bone mineral density (BMD) and body composition [perivertebral muscular mass index (PMI), skeletal muscular mass index (SMI) and muscular fat fraction] were evaluated through QCT. Musculoskeletal perfusion parameters were measured by CTP. Differences in these parameters between (or among) two (or three) groups (grouped by BMD, SMI, and TNM staging) were analyzed.

**Results:**

There were significant differences in PMI and muscular fat fraction among normal (*n* = 30), osteopenia (*n* = 43), and osteoporosis (*n* = 23) groups (both *P* < 0.001). Blood flow (*r* = 0.336, *P* = 0.001; adjusted for age and gender, r = 0.383, *P* < 0.001), blood volume (*r* = 0.238, *P* = 0.011; adjusted for age and gender, *r* = 0.329, *P* = 0.001), and flow extraction product (*r* = 0.217, *P* = 0.034; adjusted for age and gender, *r* = 0.320, *P* = 0.002) vaules of vertebral perfusion showed positive correlation with BMD. However, the relationships between PMI and perfusion parameters of perivertebral muscle were not significant. No significant differences were found in musculoskeletal mass and perfusion parameters between different TNM staging.

**Conclusions:**

The changes of bone mass and perivertebral muscular mass in patients with gastrointestinal malignancy are synchronous. Decreased vertebral bone mass is accompanied with reduced perivertebral muscular mass, increased muscular fat, and decreased bone perfusion. However, the changes of perfusion in vertebra and perivertebral muscles are asynchronous. Musculoskeletal mass and perfusion have no correlation with TNM staging of the patients with gastrointestinal malignancy.

**Trial registration:**

SHSY-IEC-4.1/20–242/01 (Registered 09–12-2020, Retrospectively registered).

**Supplementary Information:**

The online version contains supplementary material available at 10.1186/s12891-022-05288-8.

## Background

With the development of aging population in China, the problem of musculoskeletal system is becoming an important public health problem. Osteoporosis and sarcopenia are common in the elderly and associated with significant morbidity and mortality. Some studies have shown that osteoporosis and sarcopenia are closely related to the prognosis and quality of life of cancer patients [1, 2].

Recently, the hypothesis that reduced bone marrow perfusion is closely related to compromised bone mineral density (BMD) has attracted researchers’ attention [3]. Some studies have found that decreased BMD is accompanied by reduced bone marrow perfusion, furthermore, the reduced bone marrow perfusion may be helpful to predict the early development of osteoporosis [4]. Sarcopenia is closely related with osteoporosis. They share common risk factors and biological pathways [5]. To our knowledge, few studies investigated the relationship between musculoskeletal mass and perfusion in the patients with gastrointestinal malignancy. For this population, whether musculoskeletal mass and perfusion correlated with their TNM staging was also still unclear.

CT perfusion (CTP) is a kind of non-invasive imaging technology, which can evaluate the perfusion of parenchyma organs and tumors. A series of perfusion parameters can be obtained after mathematical model translation and image pseudo-color processing. For the patients with gastrointestinal malignancy who require abdominal contrast-enhance CT examination before treatment, the information of lumbar BMD, body composition, perfusion parameters, and images for diagnosis can be obtained at the same time through quantitative computer tomography (QCT) and CTP, followed by contrast-enhanced CT examination. The purpose of this preliminary study was to investigate the correlation between musculoskeletal mass and perfusion using QCT and CTP in patients with gastrointestinal malignancy.

## Methods

### Patient population

From May 2019 to February 2021, a total of 96 patients with gastrointestinal malignancy were included in this study in Shanghai Tenth People’s Hospital. The TNM staging was performed in all subjects according to the 8th edition AJCC gastric and colorectal cancer staging manual [6]. In this research, TNM I-II stages were defined as low-grade group and TNM III-IV stages were defined as high-grade group.

The inclusion criteria were as follows: (1) patients diagnosed with gastrointestinal malignancy with preoperative CT scan and endoscopy; (2) patients required abdominal contrast-enhanced CT examination; (3) age > 18 year-old; and (4) patients were volunteered for a lumbar QCT examination.

Exclusion criteria were as follows: (1) patients were suffering from cachexia or long-term digestive tract dysfunction [7]; (2) patients received special treatment including surgical operation, chemotherapy, radiotherapy, and immunotherapy; (3) patients had any history of metabolic disease or taking drugs that may affect bone metabolism, such as hormone drugs, vitamin D, bisphosphonate, etc.; (4) patients were relying on enteral or external nutrition; (5) patients with previous spinal surgery; (6) patients had contraindications of CT or CTP examinations; and (7) poor-quality CT images affected observation and measurement. This prospective study was approved by the ethics committee of Shanghai Tenth People’s Hospital. Written informed consent was obtained from all subjects.

### Imaging techniques

#### Measurement of BMD and body composition

**Fig. 1:**
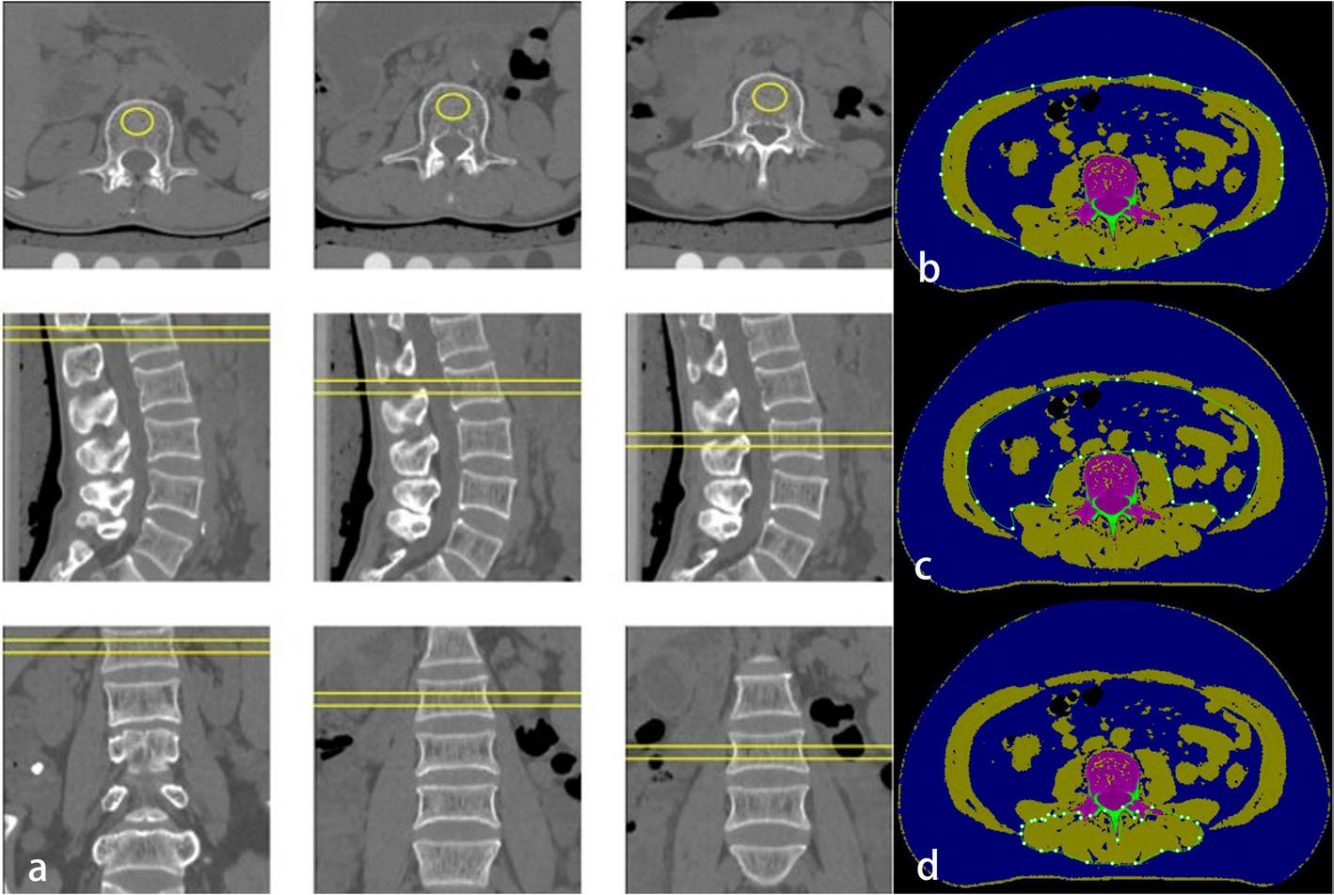
The measurement methods of bone mineral density (BMD) (**a**), area of soft tissue (**b**), area of the viscera (**c**), and perivertebral muscular mass and fat fraction (**d**). The average volumetric BMD of L1~L3 vertebral bodies was calculated as BMD value. Total skeletal muscular mass was calculated by subtracting the area of the viscera from the area of soft tissue on the axial image of the central level of L3. The measurement of perivertebral muscular mass and fat fraction was performed within the range manually drawn around perivertebral muscles on the axial image of the central level of L3

All subjects underwent spine QCT scans with a dual-source CT (Somatom Force, Siemens Healthcare, Forchheim, Germany) and a solid-state CT calibration phantom (Mindways software Inc., Austin, TX, USA). The scanning range included the vertebral bodies of lumbar vertebrae 1–5 (L1 ~ L5). QCT examination was performed by the following parameters: tube voltages 120 kV, tube current 125 mAs, slice thickness 5 mm, reconstructed slice thickness 1.5 mm. The images were transferred to a QCT PRO workstation and analyzed by QCT PRO software (Mindways software Inc., Austin, TX, USA). Regions of interest (ROI) were placed in the center of L1 ~ L3 vertebral body on axial, sagittal and coronal images. The average volumetric BMD of L1 ~ L3 vertebral bodies was calculated as BMD value (Fig. 1). The subjects were categorized into normal group (BMD ≥ 120 mg/cm^3^), osteopenia group (80 mg/cm^3^ ≤ BMD < 120 mg/cm^3^), and osteoporosis group (BMD < 80 mg/cm^3^) according to ACR criteria [8].

We measured the cross-section areas (CSAs) of total skeletal muscles, perivertebral muscles (including multifidus muscles and erector spinae muscle) and intramuscular fat within perivertebral muscles. To do this, all voxels were divided into adipose [-190 ~ -30 hounsfield units (Hu)], soft tissue (-29 ~ 150 Hu), and bone (> 150 Hu) according to the CT value on the axial image of the central level of L3 [9, 10]. The fascial boundary was manually modified and then the software calculated the CSA of total skeletal muscles, perivertebral muscles, and intramuscular fat at L3 level [11] (Fig. 1). The CSAs of perivertebral muscles and total skeletal muscles at L3 level were normalized to height squared (cm^2^/m^2^) to acquire perivertebral muscular mass index (PMI) and skeletal muscular mass index (SMI). The subjects were categorized into normal group and low muscular mass group according to previous studies [12]. The muscular fat fraction [CSA of intramuscular fat/ (CSA of intramuscular fat + CSA of perivertebral muscles) × 100%] was calculated [13].

#### Measurement of musculoskeletal perfusion

After QCT scan which could be used as that of abdominal plain CT, a bolus injection of nonionic iodinated contrast agent (Iodixanol Injection; 320 mg I/ml; Jiangsu Hengrui Pharmaceuticals Co., Ltd., Jiangsu, China; dose, 1.5 ml/kg body weight) was injected into the antecubital vein at 5.0 ml/s followed by 50 ml of saline flush at the same rate via high pressure injection (Ulrich XD8000; Germany). Consecutive dynamic contrast-enhanced CT (CTP) scan was performed within the range of L3 vertebra after a 5 s-delay from the start of injection. The CTP examination was performed by the following parameters combined with Care Dose 4D technique: tube voltages 80 kV, reference tube current 125 mAs, slice thickness 3 mm, slice interval 3 mm, total scanning time 45 s, scanning range 114 mm. Dual-phase contrast-enhanced CT scan (venous and delay phases; 60 s and 120 s-delay from the start of injection, respectively) of the abdomen was performed after CTP scan to obtain abdominal post-contrast CT images.

**Fig. 2:**
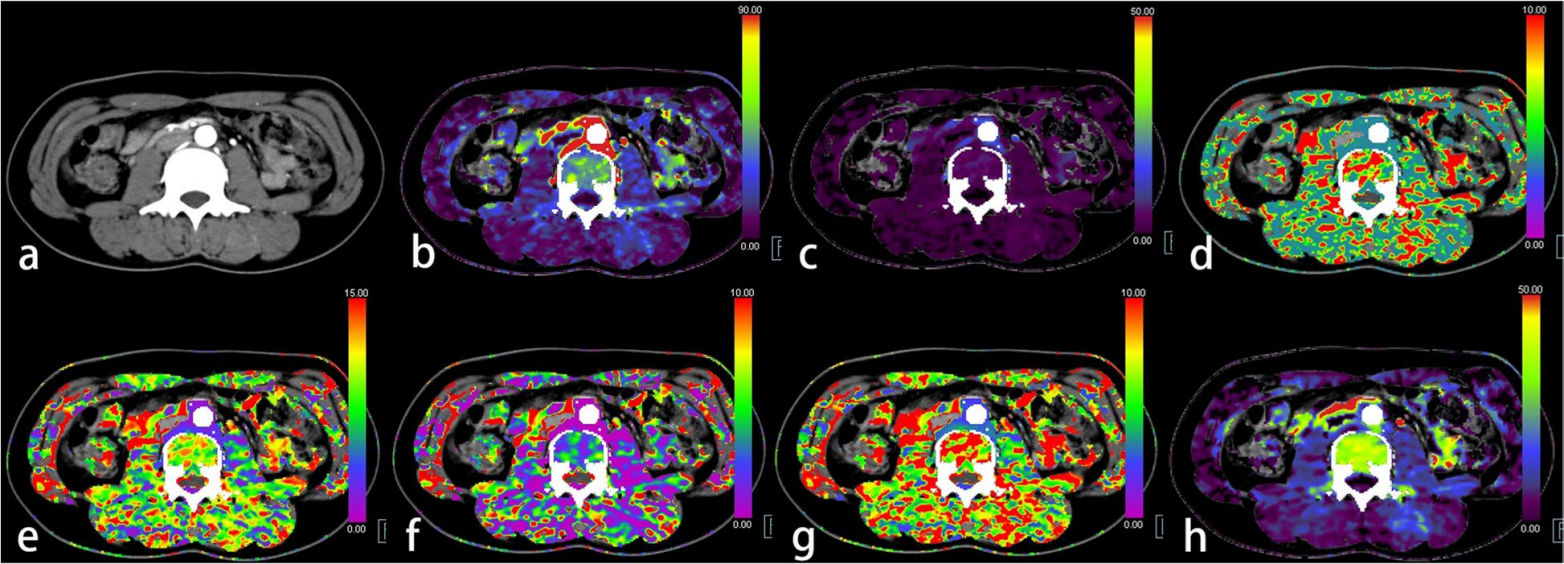
A 25-year-old female with normal bone mineral density, normal muscular mass, and low-grade sigmoid colon cancer (T_3_N_0_M_0_). **a** Axial abdominal contrast-enhanced CT image located at the central level of the vertebral body of lumbar 3. **b** The blood flow (BF) map of the CT perfusion (CTP). **c** The blood volume (BV) map. **d** The mean transit time (MTT) map. **e** The time to drain (TTD) map. **f** The time to start (TTS) map. **g** The time to maximum (T_max_) map. **h** The flow extraction product (FE) map

**Fig. 3:**
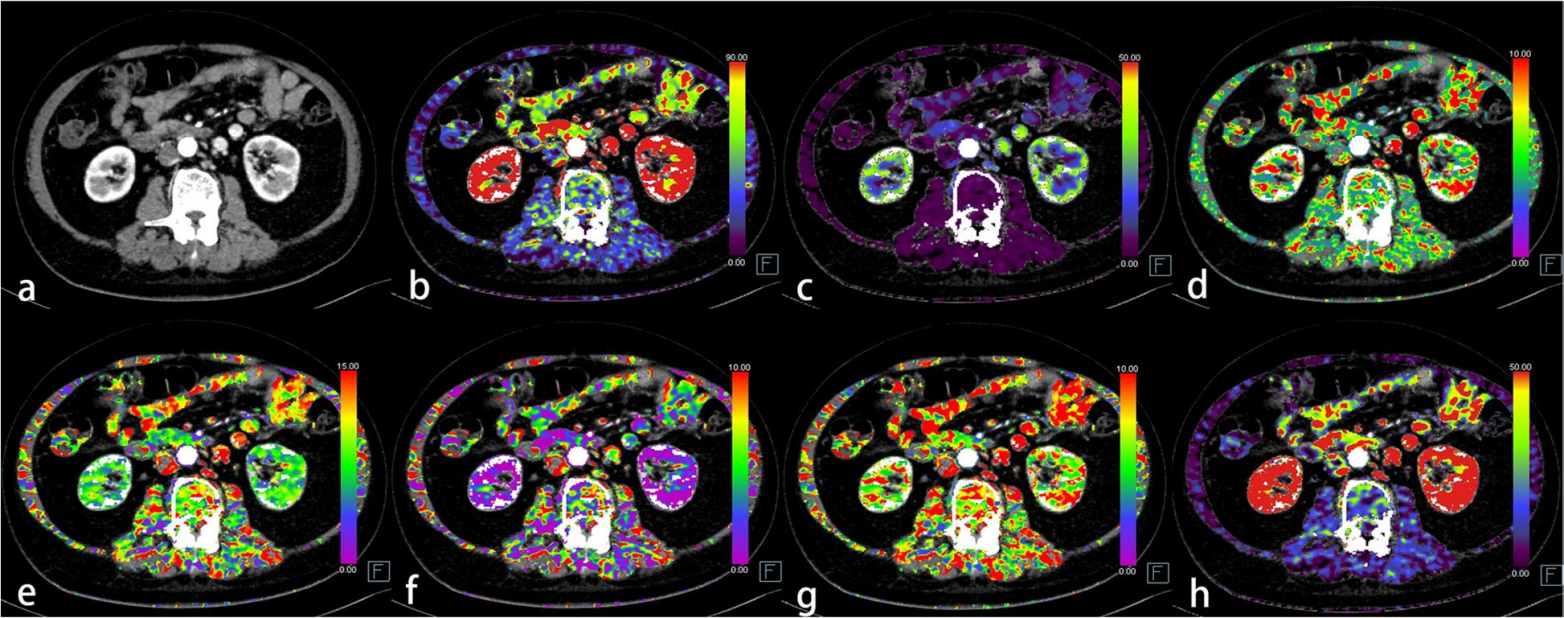
A 73-year-old male with normal bone mineral density, normal muscular mass, and high-grade sigmoid colon cancer (T_1_N_1_M_0_). **a** Axial abdominal contrast-enhanced CT image located at the central level of the vertebral body of lumbar 3. **b** The blood flow (BF) map of the CT perfusion (CTP). **c** The blood volume (BV) map. **d** The mean transit time (MTT) map. **e** The time to drain (TTD) map. **f** The time to start (TTS) map. **g** The time to maximum (T_max_) map. **h** The flow extraction product (FE) map

**Fig. 4:**
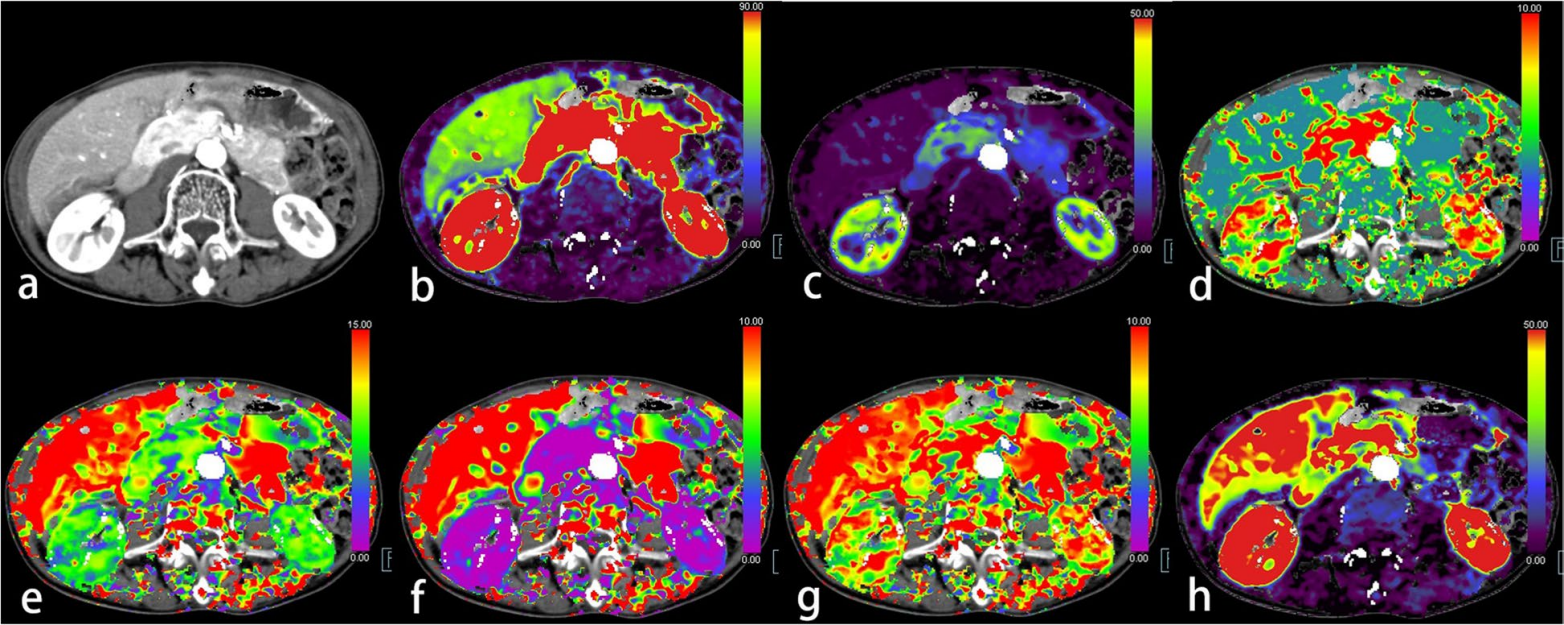
A 66-year-old male with osteoporosis, low muscular mass, and high-grade sigmoid rectal cancer (T_4a_N_1_M_1a_). **a** Axial abdominal contrast-enhanced CT image located at the central level of the vertebral body of lumbar 3(L3). **b** The blood flow (BF) map of the CT perfusion (CTP). **c** The blood volume (BV) map. **d** The mean transit time (MTT) map. **e** The time to drain (TTD) map. **f** The time to start (TTS) map. **g** The time to maximum (T_max_) map. **h** The flow extraction product (FE) map

The images were transferred to a workstation (Syngo.via, version VB10B; Siemens Healthcare, Germany) and processed offline. Processing steps included automatic four-dimensional noise and motion correction, automatic bone removal, vessel definition and generation of time-attenuation curve by placing ROI in the aorta abdominals. The ROI of bone was drawn manually on the axial image of the central level of L3 along the subcortex margins. The perivertebral muscles were selected to draw the maximum round ROI manually on the same level of L3.The perfusion parameters of the bone [blood flow (BF_B_), blood volume (BV_B_), mean transit time (MTT_B_), time to start (TTS_B_), time to drain (TTD_B_), time to maximum (T_max_B_) and flow extraction product (FE_B_)] and perivertebral muscles (BF_M_, BV_M_, MTT_M_, TTS_M_, TTD_M_, T_max_M_ and FE_M_) were measured and the average perfusion values of bilateral perivertebral muscles were taken (Figs. 2, 3 and 4; Table 1).

**Table 1:** Definition of the parameters of CT perfusion

Abbreviations	Variable	Description	Standard unit
BF	Blood flow	the blood volume flowing across the given volume of tissue per minute	ml/(100 mL·min)
BV	Blood volume	the total blood volume flowing across the given volume of tissue	ml/100 ml
MTT	Mean transit time	the mean time taken for blood to flow from the artery to the vein	s
TTS	Time to start	the time to the start of enhancement in a given region	s
TTD	Time to drain	the time from the maximum enhancement to the end of enhancement in a given region	s
T_max_	Time to maximum	the time from the start to the maximal enhancement in a given region	s
FE	Flow extraction product	the blood volume from the intravascular to the extravascular space flowing across the given volume of tissue per minute	ml/(100 mL·min)

### Statistical analysis

All statistical analyses were performed by statistical software (SPSS 25.0; SPSS, Chicago, III). To calculate inter- and intra-observer error, 20 subjects were re-evaluated by two experienced radiologists (radiologists Zhu J and Tang G, who had 17 and 38 years of experience in musculoskeletal radiology, respectively) with a 7-day interval. Intraclass correlation coefficient (ICC) test was used to calculate inter- and intra-observer agreement. The normality analysis of continuous data was performed by the Shapiro–Wilk test. The descriptive statistics of normal variables were expressed as mean ± standard deviation. The differences among different groups in terms of normal variables were compared through One-way ANOVA. The descriptive statistics of non-normal variables were expressed as median (interquartile range). The differences among different groups in terms of non-normal variables were compared through Mann–Whitney U test or Kruskal–Wallis H test. Multiple comparisons were adjusted with the Bonferroni test. The differences between categorical variables were tested by the Chi square test. The Pearson’s and Spearman’s correlation coefficient were applied for normal and non-normal distribution variables, respectively. *P* value < 0.05 was considered statistically significant.

## Results

### Subjects

Totally 96 patients [mean age 66.31 ± 10.59 years, range 25–90 years; 61/96 (63.5%) male, mean age 66.18 ± 9.72 years, range 38–90 years; 35/96 (36.5%) female, mean age 66.54 ± 12.11 years, range 25–87 years] participated in this study, including 21 patients with gastric cancer and 75 patients with colorectal cancer. Most female patients (33/35, 94.3%) in this study were in the menopausal state.

### Inter- and intra-observer agreements

Inter- and intra-observer correlation tests presented good agreements varying from 0.789 to 0.989 and from 0.815 to 0.989, respectively (Table 2).

**Table 2:** Inter- and intra-observer agreement

ICC/Parameter	BMD	PMI	FF	BF_B_	BV_B_	MTT_B_	TTD_B_	TTS_B_	T_max_B_	FE_B_	BF_M_	BV_M_	MTT_M_	TTD_M_	TTS_M_	T_max_M_	FE_M_
Inter-observer	0.988	0.983	0.989	0.871	0.943	0.963	0.906	0.897	0.789	0.970	0.906	0.957	0.939	0.904	0.941	0.885	0.927
Intra-observer	0.984	0.970	0.989	0.860	0.926	0.954	0.924	0.908	0.815	0.961	0.842	0.915	0.936	0.835	0.894	0.830	0.905

*ICC* Intraclass correlation coefficient, *BMD* bone mineral density, *PMI* perivertebral muscular mass index, *FF* fat fraction, *BF*_*B*_ blood flow of lumbar vertebra, *BV*_*B*_ blood volume of lumbar vertebra, *MTT*_*B*_ mean transit time of lumbar vertebra, *TTD*_*B*_ time to drain of lumbar vertebra, *TTS*_*B*_ time to start of lumbar vertebra, *T*_*max_B*_ time to maximum of lumbar vertebra, *FE*_*B*_ flow extraction product of lumbar vertebra, *BF*_*M*_ blood flow of perivertebral muscles, *BV*_*M*_ blood volume of perivertebral muscles, *MTT*_*M*_ mean transit time of perivertebral muscles, *TTD*_*M*_ time to drain of perivertebral muscles, *TTS*_*M*_ time to start of perivertebral muscles, *T*_*max_M*_ time to maximum of perivertebral muscles, *FE*_*M*_ flow extraction product of perivertebral muscles

### Correlation between musculoskeletal mass and perfusion

A total number of 30 normal patients, 43 osteopenic patients and 23 patients with osteoporosis patients participated in this study. There were significant differences in BF_B_, BV_B_, TTD_B_, TTS_B_, FE_B_, BV_M_, TTS_M_, and FE_M_ (all *P* < 0.05; Table 3) among normal, osteopenia, and osteoporosis groups, while no significant difference was existed in other musculoskeletal perfusion parameters (all *P* > 0.05; Table 3). Also, there were significant differences in PMI and muscular fat fraction among normal, osteopenia, and osteoporosis groups (both *P* < 0.001; Table 4). BF_B_ (*r* = 0.336, *P* = 0.001;adjusted for age and gender, *r* = 0.383, *P* < 0.001), BV_B_ (*r* = 0.238, *P* = 0.011; adjusted for age and gender, *r* = 0.329, *P* = 0.001) and FE_B_ (*r* = 0.217, *P* = 0.034; adjusted for age and gender, *r* = 0.320, *P* = 0.002) showed positive correlation with BMD. The relationship between BMD and other musculoskeletal perfusion parameters (MTT_B,_ TTD_B_, TTS_B_, T_max_B,_ BF_M_, BV_M_, MTT_M_, TTD_M_, TTS_M_, T_max_M_, and FE_M_) were not significant (*r* = 0.065, 0.044, -0.024, 0.073, 0.094, 0.091, 0.035, -0.130, -0.107, -0.085, and 0.098, respectively; all *P* > 0.05).

**Table 3:** Comparison of musculoskeletal perfusion parameters between patients with different BMD

Parameter	BMD groups			*P* value
	Normal (*n* = 30)	Osteopenia (*n* = 43)	Osteoporosis (*n* = 23)	
BF_B_ [ml/(100 mL·min)]	34.93(20.89)	28.35(11.50)	24.27(10.54)^a^	0.002
BV_B_ (ml/100 ml)	2.59(2.18)	2.49(1.93)	1.79(1.66)^a^	0.013
MTT_B_ (s)	5.03(1.98)	5.48(2.01)	4.90(1.45)	0.068
TTD_B_ (s)	10.31(1.98)	9.38(1.67)	10.71(2.63)	0.031
TTS_B_ (s)	4.97(3.17)	3.94(2.25)	5.76(3.46)^b^	0.016
T_max_B_ (s)	7.95(0.96)	7.69(1.31)	8.10(1.61)	0.453
FE_B_ [ml/(100 mL·min)]	18.86(13.17)	20.17(10.39)	14.22(14.99)^a^	0.026
BF_M_[ml/(100 mL·min)]	12.59(8.30)	12.95(8.50)	8.99(10.68)	0.053
BV_M_(ml/100 ml)	0.88(0.81)	0.92(0.95)	0.60(0.76)^b^	0.040
MTT_M_(s)	4.59(0.98)	4.68(1.22)	4.42(0.62)	0.052
TTD_M_(s)	8.64(1.70)	8.64(1.40)	8.93(2.17)	0.282
TTS_M_(s)	4.54(1.67)	4.30(2.16)	4.99(1.67)	0.030
T_max_M_(s)	6.68(1.33)	6.80(0.91)	6.78(1.20)	0.894
FE_M_[ml/(100 mL·min)]	4.45(5.46)	5.69(5.99)	3.29(3.68)^b^	0.045

*BF*_*B*_ blood flow of lumbar vertebra, *BV*_*B*_ blood volume of lumbar vertebra, *MTT*_*B*_ mean transit time of lumbar vertebra, *TTD*_*B*_ time to drain of lumbar vertebra, *TTS*_*B*_ time to start of lumbar vertebra, *T*_*max_B*_ time to maximum of lumbar vertebra, *FE*_*B*_ flow extraction product of lumbar vertebra, *BF*_*M*_ blood flow of perivertebral muscles, *BV*_*M*_ blood volume of perivertebral muscles, *MTT*_*M*_ mean transit time of perivertebral muscles, *TTD*_*M*_ time to drain of perivertebral muscles, *TTS*_*M*_ time to start of perivertebral muscles, *T*_*max_M*_ time to maximum of perivertebral muscles, *FE*_*M*_ flow extraction product of perivertebral muscles

^a^Compared with normal BMD group, *P* < 0.05

^b^Compared with osteopenia group, *P* < 0.05

**Table 4:** Comparison of PMI, muscular fat fraction, and clinical characteristics between patients with different BMD

characteristics	BMD groups			χ^2^	*P* value
	Normal (*n* = 30)	Osteopenia (*n* = 43)	Osteoporosis (*n* = 23)		
Age	60.60 ± 11.62	66.05 ± 7.97^a^	74.26 ± 8.64^ab^	13.773	< 0.001
PMI (cm^2^/m^2^)	16.64 ± 2.81	14.61 ± 2.49^a^	13.03 ± 3.37^a^	10.991	< 0.001
Muscular fat fraction (%)	9.37 ± 4.13	14.37 ± 6.77^a^	17.98 ± 11.39^a^	8.836	< 0.001
Gender				6.136	0.047
Male	24	26	11		
Female	6	17	12		
TNM staging				2.854	0.240
Low-grade	14	23	16		
High-grade	16	20	7		

*PMI* perivertebral muscular mass index, *BMD* bone mineral density

^a^Compared with normal BMD group, *P* < 0.05

^b^Compared with osteopenia group, *P* < 0.05

When grouped by gender, significant differences still existed in BF_B_, BV_B_ and FE_B_ among normal, osteopenia, and osteoporosis in male group (*P* = 0.012, 0.006, and 0.006, respectively). There was positive correlation between BMD and BF_B_, BV_B_ and FE_B_ (*r* = 0.342, 0.334, and 0.326, respectively; *P* = 0.007, 0.002, and 0.010, respectively). While there were no significant differences in all perfusion parameters among normal, osteopenia, and osteoporosis in female group (all *P* > 0.05). BMD and BF_B_ still showed positive correlation in female group (*r* = 0.407, *P* = 0.015).

When grouped by tumor staging, there were no significant differences in the perfusion parameters among normal, osteopenia, and osteoporosis in the low-grade group (all *P* > 0.05). BMD and BF_B_ showed positive correlation in low-grade group (*r* = 0.281, *P* = 0.041). In the high-grade group, significant differences existed in BF_B_, BV_B_, TTS_B_, and FE_B_ (*P* = 0.008, 0.005, 0.010 and 0.003, respectively) among normal, osteopenia, and osteoporosis. BF_B_ (*r* = 0.389, *P* = 0.010) and BV_B_ (*r* = 0.321, *P* = 0.036) showed positive correlations with BMD in high-grade group, respectively.

#### Correlation among the TNM staging, musculoskeletal mass and musculoskeletal perfusion

There were totally 53 patients in low-grade group and 43 patients in high-grade group. BMD was significantly lower in female than that in male (*P* = 0.047; Table 4). Similarly, muscular mass was significantly lower in female (17 patients with low muscular mass) than that in male (17 patients with low muscular mass) (*P* = 0.041). However, no significant difference existed in musculoskeletal mass between different TNM staging (*P* = 0.240 and 0.741, for BMD and muscular mass, respectively; Tables 4 and 5). No significant difference in musculoskeletal perfusion parameters was found between different TNM staging (all *P* > 0.05; Table 6).

**Table 5:** Comparison of muscular mass in patients with different TNM staging

Group	Normal muscular mass	Low muscular mass	χ^2^	*P* value
Low-grade	35	18	0.109	0.741
High-grade	27	16

**Table 6:** Comparison of musculoskeletal perfusion parameters between patients in different TNM staging

Parameter	TMN staging		*P* value
	Low-grade (*n* = 53)	High-grade (*n* = 43)	
BF_B_ [ml/(100 mL·min)]	27.94(13.57)	30.13(14.61)	0.290
BV_B_ (ml/100 ml)	2.45(1.90)	2.55(1.38)	0.677
MTT_B_ (s)	5.28(1.94)	5.10(1.30)	0.363
TTD_B_ (s)	9.79(2.41)	10.25(1.89)	0.664
TTS_B_ (s)	4.03(3.34)	4.82(2.78)	0.309
T_max_B_ (s)	7.99(1.22)	7.79(1.27)	0.591
FE_B_ [ml/(100 mL·min)]	19.60(12.80)	17.20(9.67)	0.874
BF_M_[ml/(100 mL·min)]	11.02(9.53)	11.94(9.86)	0.721
BV_M_(ml/100 ml)	0.79(1.05)	0.83(0.66)	0.796
MTT_M_(s)	4.64(1.18)	4.62(0.74)	0.956
TTD_M_(s)	8.31(1.43)	8.90(1.75)	0.114
TTS_M_(s)	4.32(2.11)	4.76(1.80)	0.112
T_max_M_(s)	6.59(1.12)	6.84(0.91)	0.190
FE_M_[ml/(100 mL·min)]	4.10(6.59)	5.30(4.32)	0.363

*BF*_*B*_ blood flow of lumbar vertebra, *BV*_*B*_ blood volume of lumbar vertebra, *MTT*_*B*_ mean transit time of lumbar vertebra, *TTD*_*B*_ time to drain of lumbar vertebra, *TTS*_*B*_ time to start of lumbar vertebra, *T*_*max_B*_ time to maximum of lumbar vertebra, *FE*_*B*_ flow extraction product of lumbar vertebra, *BF*_*M*_ blood flow of perivertebral muscles, *BV*_*M*_ blood volume of perivertebral muscles, *MTT*_*M*_ mean transit time of perivertebral muscles, *TTD*_*M*_ time to drain of perivertebral muscles, *TTS*_*M*_ time to start of perivertebral muscles, *T*_*max_ M*_ time to maximum of perivertebral muscles, *FE*_*M*_ flow extraction product of perivertebral muscles

#### Correlation among muscular perfusion, PMI and fat fraction

PMI showed strong negative correlation with muscular fat fraction (*r* = -0.622, *P* < 0.001, adjusted for age and gender *r* = -0.462, *P* < 0.001). However, there was no significant correlation between PMI and perfusion parameters of perivertebral muscles (*r* = -0.002, -0.035, -0.158, -0.101, 0.036, -0.174, -0.087, respectively; all *P* > 0.05), and also between fat fraction and perivertebral muscle perfusion parameters (*r* = 0.037, 0.025, 0.050, 0.038, 0.009, 0.052, 0.018, respectively; all *P* > 0.05).

## Discussion

CTP is seldom to evaluate musculoskeletal system. MR perfusion is used in most studies about musculoskeletal system perfusion, mainly due to its being free of X-Ray radiation, but the unsatisfactory acquisition speed, the high cost and the poor patient’s compliance limited its clinical application. For patients with gastrointestinal malignancy who need abdominal contrast-enhanced CT examination, the information of lumbar BMD, body composition, perfusion parameters, and images for diagnosis can be obtained simultaneously through QCT and CTP, followed by contrast-enhanced CT.

In our study, lumbar BMD and muscular mass differed between the two genders, with females showing less musculoskeletal mass than males. Most female patients in this study were in menopause. Estradiol is closely related to BMD and plays a positive role in bone remodeling by inhibiting the activity of osteoclasts and promoting the synthesis of 1,25-dihydroxyvitamin D3 [14]. Lean mass was a strong factor associated with BMD. Previous studies had shown that the decrease of weight may be a major risk factor for bone loss in the elderly [15]. Kieron et al. [16] found that the female gender was an independently associated factor of sarcopenia which was consistent with our research. Higher prevalence of osteopenia was related to the weakness of the flexor and extensor muscles of the lower extremities in women [17]. Musculoskeletal mass is closely related to each other, which has a great influence on the quality of life of the elderly, especially the female cohort [18].

Our research showed that as BMD decreased, there was a corresponding reduction of BF and BV, suggesting blood flow velocity and blood volume of the vertebral body decreased. Ou-yang et al. [19] using stratified sampling randomly enrolled 186 healthy people and measured average CTP parameters and BMD of the third lumbar vertebrae. They found that the CTP parameters (BV and BF) showed strong positive correlations with BMD (*r* = 0.806 and 0.685, respectively), which concurred with our research. Many possible mechanisms may lead to this phenomenon. Some clinical studies had shown that female patients with osteopenia or osteoporosis were more common to get coronary artery diseases, ischemic stroke or peripheral arterial diseases [20]. Ma et al. [21] found that quantitative DCE-MRI parameters significantly reduced in patients with lower BMD. Griffith et al. [3] suggested that increased marrow fat content made a great contribution to reduced bone marrow perfusion. Zhu et al. [4] considered that enhanced vasoconstriction, increased marrow adipose tissue and fibrosis, and decreased microvessel density may aggravate ischemia in the bone marrow of rat osteoporosis model. Previous clinical study revealed that the utilization rate of nitric oxide decreased in osteoporosis patients, which resulted in vascular endothelial dysfunction accompanied by a decrease of bone marrow perfusion [22]. FE was used to reflect vascular permeability, which was reduced in the bone marrow with the decrease of BMD in our study, indicating the decrease of vascular permeability. The same phenomenon was found in the male group and the high-grade patients, but not obvious in female and low-grade patients. This might be related to the relatively small proportion of female in our study.

At present, there were rare studies on the relationship among muscular mass, muscular fat fraction and muscular perfusion using non-invasive imaging methods. Our study showed that there was no significant correlation among muscular mass, and muscular fat fraction were not related to and muscular perfusion. Similar to osteoporosis, with the decrease of BMD, the muscle mass decreased and the muscular fat fraction increased. However, unlikely to vertebral body, the increase of muscular fat fraction didn’t influence muscle perfusion in our study. Griffith et al. [3] reported that the reduced perfusion of hip osteoporotic bone was confined to the bone marrow, rather than the influence of circulatory disturbance, without reduction of perfusion in the adductor muscle of the thigh at the same time. This result was consistent with our study. Although vertebral body and perivertebral muscle are both supplied by the small branches of abdominal aorta, the changes of perfusion are asynchronous [23, 24]. This phenomenon maybe related to the different microenvironment of the microvessels of bone and muscle. Picca et al. [25] found that increased levels of oxidative stress and damage of mitochondria induced the decrease of muscle mass, this may also be an important mechanism for muscular mass reduction without a decrease in muscle perfusion.

It was worth noting that no significant difference existed in musculoskeletal mass and perfusion between different TNM staging in our study. The reduction of musculoskeletal mass in patients with malignant tumors was often accompanied by cachexia which appeared in the later stage [26]. However, to investigate the relationship between musculoskeletal mass and perfusion in untreated cases with gastrointestinal malignancy, patients with cachexia or received special treatment (including surgical operation, chemotherapy, radiotherapy, and immunotherapy, et al.) were excluded. Owing to the relatively small sample in our study, a larger multicenter study to verify our findings is needed.

There were several limitations in our study. First, the total number of subjects was small, especially in osteoporosis, female, and high-grade tumor groups. Due to the strict exclusion criteria, patients with high-grade tumor developing cachexia could not meet the inclusion criteria. As for the small proportion of female, males suffered a higher risk of gastrointestinal malignancy than females according to a Chinese epidemiological study of cancer patients [27]. Second, the condition of menopause may be an influence on our research. More detailed analysis will be carried out in our further study with larger sample size. Furthermore, the patients received radiation to evaluate musculoskeletal perfusion using CTP. In order to reduce the radiation, the range of scanning was limited. At the same time, the automatic real-time dose adjustment technology of Siemens CARE Dose 4Dtechnology was adopted. The principle of CARE Dose 4D technique was modulation of real-time z-axis tube current and real-time angular dose. Also CARE Dose 4D technique had been confirmed to reduce radiation dose in the previous study [28]. In our study, the total effective radiation dose for each subject was relatively low.

## Conclusion

The changes of bone mass and perivertebral muscular mass at L3 level in patients with gastrointestinal malignancy are synchronous. Lumbar BMD and muscular mass differed between the two genders. Female has less musculoskeletal mass than male. Decreased vertebral bone mass is accompanied with reduced perivertebral muscular mass, increased intramuscular fat and decreased bone perfusion. The changes of perfusion in vertebra and perivertebral muscles at L3 level are asynchronous, which implies that reduced perfusion in osteoporosis may be confined to the bone. However, musculoskeletal mass and perfusion have no correlation with TNM staging of the patients with gastrointestinal malignancy.

## Supplementary Information

**Additional file 1.** Total skeletal muscular mass (cm²).

## Data Availability

All data generated or analysed during this study are included in this published article and its supplementary information files.

## Abbreviations

BF: Blood flow
BMD: Bone mineral density
BV: Blood volume
CSA: Cross-section areas
CTP: CT perfusion
FE: Flow extraction product
Hu: Hounsfield units
ICC: Intraclass correlation coefficient
MTT: Mean transit time
PMI: Perivertebral muscular mass index
QCT: Quantitative computer tomography
ROI: Regions of interest
SMI: Skeletal muscular mass index
T_max_: time to maximum
TTD: Time to drain
TTS: Time to start
