# Correction: Correlation between musculoskeletal mass and perfusion in patients with gastrointestinal malignancy: a preliminary study based on quantitative CT and CT ﻿perfusion

**DOI:** 10.1186/s12891-022-05386-7

**Published:** 2022-05-10

**Authors:** Rui Ji, Lin Zhang, Yongju Shen, Rui Tang, Yun Tu, Guangyu Tang, Jingqi Zhu

**Affiliations:** Department of Radiology, Shanghai Tenth People’s Hospital, Tongji University School of Medicine, 301 Middle Yanchang Road, Shanghai, 200072 China

**Correction to: BMC Musculoskelet Disord 23, 334 (2022)**

**https://doi.org/10.1186/s12891-022-05288-8**

Following the publication of the original article [1] the author reported that caption of Additional file 1 is incorrect. The correct caption should read:

“This table provides the original data of the submitted manuscript, which includes gender, age, height, TNM staging, and parameters of QCT and CTP.”

The original article [1] has been updated.

## Supplementary Information

**Additional file 1.** : This table provides the original data of the submitted manuscript, which includes gender, age, height, TNM staging, and parameters of QCT and CTP.
